# Exploring the physiotherapy and exercise needs and preferences of nursing home residents with dementia: A qualitative study

**DOI:** 10.1177/25424823251349166

**Published:** 2025-06-09

**Authors:** Dennis Boer, Romée Nibbering, Charlotte Schmidt, Shanty Sterke, Eefje Sizoo, Wilco Achterberg, Thea Vliet Vlieland

**Affiliations:** 1 Department of Innovation and Research, Kennemerhart, Haarlem, The Netherlands; 2 Department of Physiotherapy, 125778 University of Applied Sciences Leiden, Leiden, The Netherlands; 3 Department of Orthopedics, Rehabilitation and Physiotherapy, 4501 Leiden University Medical Center, Leiden, The Netherlands; 4 Research Centre Innovations in Care, 6985 Rotterdam University of Applied Sciences, Rotterdam, The Netherlands; 5 Department of Physiotherapy, 6994 Aafje Nursing Homes, Rotterdam, The Netherlands; 6 Department of Public Health, 6993 Erasmus University Medical Center, Rotterdam, The Netherlands; 7 Department of Neurology, 26066 Amsterdam University Medical Center, Amsterdam, The Netherlands; 8 Department of Primary Care and Public Health, 4501 Leiden University Medical Center, Leiden, The Netherlands

**Keywords:** Alzheimer's disease, care needs, exercise, long-term care, neurocognitive disorder, qualitative research

## Abstract

**Background:**

Exercise is widely employed to prevent functional decline in individuals with Alzheimer's disease and other forms of dementia. Exercise interventions are often supervised by physiotherapists, particularly in Western countries. However, adherence to exercise-based interventions remains suboptimal, particularly among nursing home residents.

**Objective:**

To investigate the needs and preferences of nursing home residents with mild to moderate dementia concerning physiotherapy and exercise interventions.

**Methods:**

Semi-structured individual interviews were conducted with 12 residents from various nursing homes who had been diagnosed with mild to moderate dementia, were proficient in Dutch, and capable of providing informed consent. Data from the interviews were analyzed using thematic analysis.

**Results:**

Four key themes were identified: perceptions of physiotherapy and preferences for physiotherapy sessions, defining physiotherapy and exercise, exercise without physiotherapist supervision, and communication. In general, residents preferred physiotherapy that incorporated exercise and guidance aimed at preserving their independent physical functioning. Physiotherapy was perceived as more intensive than general exercise classes, and not necessarily suitable for all residents. Participants indicated that, provided safety and quality were maintained, they were willing to perform exercises independently. Residents expressed a desire for their family caregivers to be kept informed about their therapy, although they largely preferred to exercise with another person.

**Conclusions:**

Nursing home residents with mild to moderate dementia expressed distinct preferences regarding physiotherapy sessions, communication, and family caregiver involvement. Addressing these preferences may improve adherence to and the effectiveness of exercise interventions. Additionally, the findings suggest a potential shift toward a more supervisory role for physiotherapists, rather than the traditional hands-on approach.

## Introduction

Physiotherapy is commonly utilized to reduce functional decline in nursing home residents with dementia. In Western countries, the proportion of nursing home residents receiving physiotherapy ranges from 10% to 67%, varying by country, ward type, and facility characteristics.^1^ Exercise constitutes a core component of physiotherapy interventions for this population.^2,3^ A comprehensive guideline offering evidence-based recommendations on exercise for nursing home residents has been developed by experts in geriatrics and exercise science.^4^ While the guideline underscores the importance of incorporating residents’ needs and preferences into physiotherapy interventions, it does not provide detailed insights into the specific nature of these needs and preferences.

Previous studies have demonstrated that nursing home residents with dementia are frequently able to articulate their needs and preferences concerning therapy and healthcare decisions.^5,6^ However, other evidence indicates that these needs are not consistently addressed, and residents with dementia are often excluded from medical decision-making processes.^7^ Such exclusion may be viewed as both ethically problematic and detrimental to adherence, as the lack of consideration for residents’ needs and preferences can compromise engagement with interventions.^8,9^ Consequently, this may reduce the overall effectiveness of the intervention.

To our knowledge, three studies have explored the perceptions of nursing home residents with dementia regarding physiotherapy or physiotherapist-guided exercise interventions.^10–12^ In one study^10^ interviews were conducted with individuals with dementia and their family caregivers, including three residents in long-term care. Although the study primarily focused on person-centered care, it highlighted the role of mutual understanding and clear communication between patients and physiotherapists.^10^ In another study,^11^ ten nursing home residents with dementia who had completed a physiotherapist-supervised high-intensity exercise program shared their experiences, emphasizing the significance of effective communication, empathy, and tailoring therapy to their individual needs. A follow-up study two years later, involving 21 nursing home residents with dementia who participated in the same exercise program, reaffirmed these findings, underscoring the importance of clear communication and the incorporation of residents’ personal preferences in physiotherapy.^12^

The aforementioned studies have underscored the importance of integrating the needs and preferences of residents with dementia into physiotherapy. However, these investigations primarily focused on the experience of person-centered care^10^ and reflections on a specific high-intensity exercise program.^11,12^ Further research is needed to explore residents’ needs and preferences concerning future physiotherapy or exercise sessions, which may aid in the design of interventions that enhance adherence. Further insights into preferences regarding the FITT-VP factors^13^ (Frequency, Intensity, Time, Type, Volume, and Progression), which constitute the fundamental elements of exercise, could inform future interventions. Additionally, a previous study identified a gap in knowledge among individuals with dementia concerning the perceived role of physiotherapists.^10^

Therefore, the objective of our study is to examine the needs and preferences of nursing home residents with mild to moderate dementia in relation to physiotherapy and associated exercise interventions.

## Methods

This study utilized a descriptive qualitative design, employing face-to-face semi-structured interviews. No *a priori* hypothesis was formulated; instead, concepts and theories were developed inductively from the collected data. Ethical approval was obtained from the medical ethics committee of the orthopedics department at Leiden University Medical Center (LUMC). The study was conducted in accordance with the ethical principles outlined in the World Medical Association's Declaration of Helsinki.^14^ The Consolidated criteria for Reporting Qualitative research (COREQ) guidelines^15^ were adhered to in both the study design and reporting.

### Setting

Participants were recruited from three nursing homes affiliated with the long-term care organizations “Kennemerhart” and “Aafje,” located in urban areas in the western part of the Netherlands. Collectively, these organizations oversee 27 nursing homes across the provinces of Noord Holland and Zuid Holland, accommodating approximately 1100 residents with dementia. The nursing homes offer small-scale care settings, consisting of private bedrooms and shared common areas designed for groups of up to 10 residents.

### Participants and sampling

Nursing home residents with dementia who were receiving physiotherapy at the time of recruitment were selected by the supervising physician based on the following criteria: a physician-confirmed diagnosis of dementia documented in the medical record, proficiency in the Dutch language, and the capacity to provide informed consent for participation in an interview, as determined by both the supervising physician and the physiotherapist.

Participants received physiotherapy in accordance with standard physiotherapy practices in Dutch nursing homes.^3,16^ The therapy primarily consists of supervised individual exercise sessions; however, group exercise sessions and manual passive mobilizations may also be utilized. The primary goals of physiotherapy are to improve mobility and reduce the risk of falls.^3^

In the Netherlands, clinicians commonly use the umbrella term “dementia” without further specifying the subtype, such as Alzheimer's disease, vascular dementia, frontotemporal dementia, or mixed-type dementia. Residents were excluded from the study if they had a non-dementia pathology significantly affecting their functional status, exhibited behavioral or communication issues that could hinder participation in an interview, were expected to experience heightened behavioral problems (e.g., agitation or distrust) when presented with an informed consent form and study protocol, or had a life expectancy of less than 12 weeks.

Physiotherapists informed eligible residents by providing an information leaflet and verbally explaining the study procedures. Residents were given a one-week consideration period before being formally invited to participate. Written informed consent was obtained from those who agreed to take part. With the residents’ permission, the principal investigator contacted their designated family caregivers to explain the study procedures and provided an information leaflet. Two versions of the information leaflet were created: one simplified version for residents with dementia, using the term “memory problems and difficulties with daily structure” instead of “dementia,” and a standard version for family caregivers containing the core study information without simplified language. A total of 24 eligible residents were contacted in two rounds (*n* = 22, *n* = 2). After conducting 10 interviews in the first round, data were coded to assess saturation (see Procedure). An additional two participants were recruited in the second round. All participants provided written informed consent.

### Procedure

The interviews were conducted between October and December 2023. Data collection continued until saturation was achieved, defined as the point at which no new codes or themes emerged, and the information gathered from subsequent interviews was consistent with previously identified patterns.^17^ Based on prior qualitative research, we anticipated saturation to occur after 12 to 15 interviews,^18,19^ which was confirmed following the 12th interview. Consequently, no additional interviews were conducted after data saturation was reached. On the day of each interview, a familiarization period was arranged to allow residents to become comfortable with the researchers, who engaged in routine activities such as sharing coffee or lunch with the residents. All interviews were conducted in the participants’ private rooms. Before commencing the interviews, the study's objectives and procedures were reviewed, and participants were reminded that there were no “right” or “wrong” answers. They were assured that their responses would remain confidential and would not affect their ongoing physiotherapy or care.

The interview guide (see the Supplemental Material) was developed based on a previous study examining the needs and preferences of informal caregivers of nursing home residents with dementia regarding physiotherapy,^19^ on the FITT-VP factors^13^ and to examine the perceived role of the physiotherapist. The guide covered four main topics: physiotherapy modalities, non-physiotherapy exercise, communication, and family involvement. Two authors (DB, RN) conducted the interviews, alternating between the roles of interviewer and observer. The observer monitored for signs of discomfort and was authorized to halt the interview if necessary. Both interviewers (DB, RN) had prior experience communicating with nursing home residents with dementia. The observer also took field notes and provided a summary of the key findings to the participant at the end of the interview for accuracy confirmation. If a pre-existing relationship with one interviewer was present, the other interviewer conducted the session; this occurred with three participants. Informal caregivers were allowed to attend the interview if desired by the resident, which happened in one instance. Interviews were audio-recorded using Microsoft Teams (version 24165.1414.2987.41), and recordings were deleted after transcription. All study data were handled confidentially in accordance with the General Data Protection Regulation (GDPR).^20^

### Data analysis

Participant characteristics were collected from the supervising physician with the participant's consent. These characteristics included age, gender, length of stay in the nursing home, number of medications, number of comorbidities, dementia subtype, religious background, and functional status, which was assessed using the Barthel Index.^21^ The Barthel Index, ranging from 0 to 20, measures independence in daily activities such as transferring, bathing, and dressing, with higher scores indicating greater independence.

Data were analyzed using thematic analysis following the methodology outlined by Braun and Clarke.^22^ Transcripts were reviewed repeatedly by the researchers (DB, RN) to ensure familiarity with the content. Relevant data were independently coded through open coding. Coding was performed using AtlaS.ti software (version 9, Scientific Software Development GmbH, Berlin). After initial independent coding, a consensus meeting was held between the two researchers to group codes through axial coding. If consensus was not achieved, a third researcher (CS) was consulted. However, in this study consensus was reached without the need for additional adjudication. In a subsequent consensus meeting, grouped codes were linked to form overarching themes. These themes were further discussed and refined in collaboration with two additional authors (CS, SS) based on their feedback.

## Results

**Figure 1.**
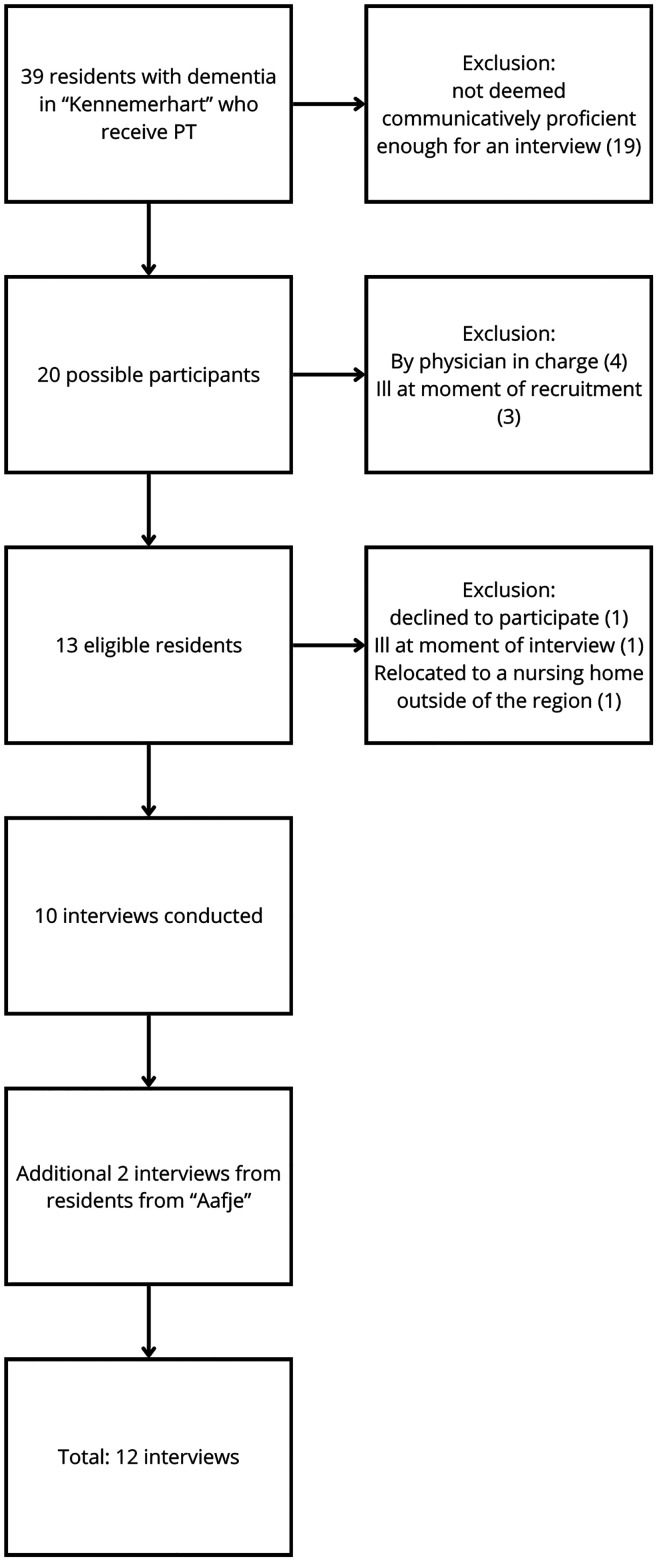
Flowchart describing the recruitment and inclusion process.

In two rounds of interviews, a total of 12 participants were interviewed (see Figure 1 for recruitment details). Baseline characteristics of the participants and interview specifics are summarized in Table 1. Participants’ ages ranged from 75 to 95 years, and their length of stay in the nursing home varied from 2 to 86 months. The number of medications taken by participants ranged from 3 to 10, while the number of comorbidities ranged from 2 to 29. Interview durations varied between 13 and 58 min, with a mean duration of 40 min. Thematic analysis yielded 82 unique codes, which were consolidated into four major themes: (1) perceptions of physiotherapy and preferences for physiotherapy sessions, (2) defining physiotherapy and exercise, (3) exercise without physiotherapist supervision, and (4) therapist-resident interaction and family involvement.

**Table 1.** Characteristics of the twelve participating nursing home resident with mild to moderate dementia.

	*n* = 12
Age (mean + SD)	83.9 (6.6)
Female (%)	7 (58%)
Length of nursing home stay in months (mean + SD)	15.3 (23.2)
Number of medications (mean + SD)	7.3 (3.4)
Number of comorbidities (mean + SD)	11.7 (8.1)
Dementia type	
Alzheimer's disease (%)	3 (25%)
Vascular (%)	2 (16.7%)
Mixed (%)	3 (25%)
Alcohol related (%)	1 (8.3%)
Not specified (%)	3 (25%)
Functional status (Barthel Index mean + SD)	12.3 (3.4)

### Theme 1: perceptions of physiotherapy and preferences for physiotherapy sessions

The most common perception of physiotherapy was that it involves exercises, particularly those focusing on walking and movement of extremities. Additionally, several participants expressed a preference for physiotherapists to offer information, guidance, and motivation during exercise sessions.

*- P11 (76-year-old male). “That is important to me. That she says: “Hey sir!, can you walk a little bit more like this?”*

Participants indicated that the goals of physiotherapy were typically aimed at maintaining or improving physical function. Some participants found it difficult to articulate if there was a specific physiotherapeutic goal, and most were unable to specify how such goals are established or who is responsible for setting them.

*- P1 (90-year-old male). “Well, yes, to be able to keep moving for as long as possible. Yes. That's the goal in the end.”*

A preference for one-on-one physiotherapy sessions was expressed by some participants. They explained that group physiotherapy could become chaotic or that they were simply accustomed to individual sessions. However, other participants preferred group sessions, noting that exercising in a group setting was more enjoyable. Regardless of the setting, the importance of receiving adequate personal attention was emphasized.

*- P10 (84-year-old female). “Yes. It's enjoyable, in a group. But when it comes to therapy, I'd prefer one-on-one.”*

A preference for a minimum of two physiotherapy sessions per week was mentioned, scheduled at consistent times and days. A card or note with the scheduled appointments was deemed useful. Residents emphasized that sessions should be of appropriate duration: too long could lead to exhaustion, while too short might leave them feeling unfulfilled, as if they had not achieved anything.

*- P12 (91-year-old female). “I think two or three times. That's what I prefer. Then you stay in a bit of a rhythm.”*

Some participants thought that physiotherapy should always be continued, even after achieving the therapy goals. Others recommended a gradual reduction of therapy once the primary health issue has been addressed.

*- P5 (88-year-old female).–“If someone wants to stay fit, you see… People who never do anything, they are as stiff as a dried fish.”*

Most participants preferred physiotherapy sessions in a gymnasium (a place in the nursing home designated for physical exercise), primarily due to the availability of exercise equipment. Participants suggested that physiotherapy could also take place in the common room, but expressed concerns about potential sensory overload in crowded spaces. Participants specifically preferred therapy locations other than their bedrooms, deeming these spaces too personal for such activities.

*- P10 (84-year-old female). “Here, you don't have any equipment. So, with him (the physiotherapist), you do. Because I also had to learn to walk between those parallel bars.”*

### Theme 2: defining physiotherapy and exercise

The general opinion among participants was that physiotherapy is primarily for individuals with health problems. It was argued that those without health issues can exercise independently without a physiotherapist's supervision. Nonetheless, it was also argued that physiotherapy should be available to everyone, as long as the therapist receives reimbursement.

*- P5 (88-year-old female). “You see, the physiotherapists who work there, they want to get their money, of course. And whether it comes from the patient or their insurance, it doesn't matter to them. As long as they get paid”.*

Participants distinguished physiotherapy from gymnastics or exercise classes, noting that physiotherapy sessions are typically more intense than gymnastics classes, which are generally considered recreational.

- *P9 (86-year-old female). “One is more feisty, the other one is more calm. That is the difference.”*

### Theme 3: exercise without physiotherapist supervision

Some participants indicated that they perform exercises independently, without the supervision of a physiotherapist. They maintained their pre-existing exercise routines established prior to admission to the nursing home and expressed no need for help from the physiotherapist. Although some participants performed exercises without direct supervision from a physiotherapist, there was a desire for guidance from a physiotherapist beforehand in the form of demonstrations of exercises or the provision of written instructions and illustrations to facilitate independent exercise sessions. Some participants did not perform unsupervised exercises because of safety concerns.

*- P12 (91-year-old female). “I'm afraid to do too much. […] Because I'm scared of falling. They just need to hold me.”*

The majority of participants felt confident exercising under the guidance of a competent non-physiotherapist. Nonetheless, most preferred not to be supervised by a family caregiver. They cited reasons such as family members being busy with their own responsibilities or living too far away. Participants preferred to spend quality time with family on enjoyable activities rather than having family members supervise or be present during structured exercise sessions.

*- P3 (83-year-old female) “They don't have time to be here. They have their work too.”*

### Theme 4: communication

Most participants expressed the preference of having a friendly, familiar and personal relationship with the physiotherapist. Some preferred to be addressed informally instead of formally. Although communication is preferred to be informal and friendly, the relationship needs to remain professional.

- *P9 (86-year-old female). I would begin by addressing him as “Sir.” However, I would likely soon ask if I could use his first name.*

Participants emphasized the importance of the physiotherapist being mindful of their limitations during physiotherapy and when communicating, such as speaking slowly and clearly and maintaining eye contact during conversations.

*- P8 (91-year-old female) “I'm not as quick anymore. I'm slower. He (the physiotherapist) shouldn’t force me to do this or that. I need to be able to do it at my own pace.”*

Most participants expressed a preference for the regular involvement of family members or informal caregivers in physiotherapy, in the form of updates on the progress of therapy sessions.

*- P12 (91-year-old female). “When the therapist comes, I also inform the children. I think it's important that they know what's going to happen.”*

## Discussion

This study identified four major themes reflecting the preferences of nursing home residents with mild to moderate dementia concerning physiotherapy and related exercise interventions. The themes included: (1) characteristics of physiotherapy, such as goals, content, dosage, and mode of delivery; (2) distinction between physiotherapy and general exercise; (3) preferences regarding supervision, whether by a physiotherapist or otherwise; and (4) communication preferences, including the resident's desired level of interaction and the involvement of family members.

To the best of our knowledge, this is the first study to specifically explore nursing home residents’ perceptions and preferences regarding physiotherapy and exercise outside the context of a specific intervention. Previous research has examined the experiences of residents who had already received physiotherapy.^10–12^ While direct comparison is challenging, certain similarities, differences, and novel insights emerged. Consistent with earlier studies, our findings underscore the importance of effective communication and an empathetic understanding of residents’ limitations. Additionally, participants in our study, similar to those in prior research, emphasized the need for appropriate support and coaching in conjunction with prescribed exercises.

Regarding exercise goals, participants in our study expressed a desire to remain physically active and delay dependency, aligning with findings from the two Scandinavian studies.^11,12^ Another noteworthy similarity is that residents in both our study and the Scandinavian studies did not express a clear preference regarding who supervises the intervention, provided that the supervisor is competent and ensures safety. This finding suggests the potential for future research exploring a model where physiotherapists adopt a more supervisory or coaching role, delegating the practical aspects of therapy to trained assistants. Given the rising healthcare costs,^23,24^ such a model could reduce financial burden while maintaining care quality. Since participants did not exhibit a strong preference for a specific supervision style, future studies could investigate whether similar levels of safety, satisfaction, and clinical outcomes can be achieved when physiotherapists primarily focus on coaching rather than direct intervention delivery.

Some discrepancies were observed when comparing the results of our study with previous literature. Participants in the Scandinavian studies specifically expressed a preference for group exercise, whereas, although our participants also reported enjoying group exercise, they preferred individual physiotherapy sessions. This difference may be attributed to the residential settings of the participants. In the Scandinavian studies, participants lived in private rooms and expressed feelings of loneliness, whereas our participants resided in small-scale, family-like wards. Although loneliness was not explicitly addressed in our study, it is plausible that residents in shared, small-scale settings experience less loneliness, which may reduce their need for social interaction during exercise activities.

The participants in the study by Hall et al.^10^ expressed dissatisfaction with the physiotherapy they received, citing factors such as insufficient resources and a lack of personalized care. This contrasts with the preferences of participants in our study, who generally expressed satisfaction with the services provided. A potential explanation for this discrepancy lies in the differing healthcare systems. A review of physiotherapy availability indicated that approximately 11% of nursing home residents in the UK receive physiotherapy, compared to 67% in the Netherlands, which likely influences satisfaction levels.^1^

Our study was conducted and reported in accordance with qualitative research guidelines. Additionally, when drafting the study protocol and implementing the methods, we carefully considered ethical literature regarding research involving individuals with dementia, which likely enhances the validity of our findings. The primary aim of our study was to explore residents’ perceptions and preferences regarding future physiotherapy interventions, with the goal of informing physiotherapy and exercise guidelines. This study has provided insights into the perspectives of nursing home residents with dementia regarding the desired delivery of exercise therapy. However, since participants were recruited while receiving physiotherapy, it cannot be ruled out that participants may have reflected more on their experiences with current physiotherapy rather than exclusively providing preferences for future interventions. No quantitative details regarding duration, intensity, or related aspects were collected, as participants found these difficult to articulate. To preserve interview rapport, the researchers intentionally chose not to explore this matter further. As noted earlier in the discussion, physiotherapy services and reimbursement practices vary significantly across countries. Although we cannot provide objective data, the reality is that many nursing homes in various countries either do not employ physiotherapists or do so under precarious conditions. Therefore, we believe the findings from this study are most applicable to physiotherapy and nursing home systems similar to those in the Netherlands.

The results of our study can inform the development of future physiotherapy and exercise interventions. Many of the preferences expressed by participants can be incorporated into practice, potentially enhancing adherence to exercise interventions. Additionally, the findings provide insight into residents’ perceptions of the role of physiotherapists in their lives. The need for understanding and personalized advice, which emerged as important to participants, is not yet a prominent aspect of physiotherapy quality standards. Given that residents are the primary recipients of physiotherapy, communication and understanding should be prioritized alongside more easily measurable outcomes, such as increases in muscle mass or improvements in balance.

In conclusion, this study explored the preferences of nursing home residents with dementia regarding physiotherapy and exercise. In addition to preferences related to therapy goals, duration, location, and individual supervision, participants expressed a desire for information from the physiotherapist and for being coached, as well as for maintaining a professional yet friendly relationship. Most participants indicated that exercise sessions could be supervised by non-physiotherapists, provided that safety and quality are ensured. While residents wanted to keep family members informed about their physiotherapy, they preferred not to exercise with them. Given that residents are key stakeholders in physiotherapy, their preferences should be considered in future studies and current practice. Incorporating these preferences has the potential to enhance both adherence to therapy and its overall effectiveness. As residents expressed no need for direct physiotherapy supervision during exercise, provided safety is maintained, the role of the physiotherapist warrants further evaluation. Shifting the physiotherapist's role from a traditional “hands-on” approach to a more supervisory or managerial role could contribute to strategies aimed at improving healthcare cost-effectiveness.

## Supplemental Material

sj-docx-1-alr-10.1177_25424823251349166 - Supplemental material for Exploring the physiotherapy and exercise needs and preferences of nursing home residents with dementia: A qualitative study Supplemental material, sj-docx-1-alr-10.1177_25424823251349166 for Exploring the physiotherapy and exercise needs and preferences of nursing home residents with dementia: A qualitative study by Dennis Boer, Romée Nibbering, Charlotte Schmidt, Shanty Sterke, Eefje Sizoo, Wilco Achterberg and Thea Vliet Vlieland in Journal of Alzheimer's Disease Reports
