# Supplementary material for: Exploring the physiotherapy and exercise needs and preferences of nursing home residents with dementia: A qualitative study
Source: J Alzheimers Dis Rep. 2025 Jun 9;9:25424823251349166. doi: 10.1177/25424823251349166 (PMC12152378; doi:10.1177/25424823251349166)
Supplement: sj-docx-1-alr-10.1177_25424823251349166 - Supplemental material for Exploring the physiotherapy and exercise needs and preferences of nursing home residents with dementia: A qualitative study [file sj-docx-1-alr-10.1177_25424823251349166.docx]

**Supplemental Material**

**Exploring the physiotherapy and exercise needs and preferences of nursing home residents with dementia: A qualitative study**

| TOPICS: |
| --- |
| 1. What is Physiotherapy? |
| We will soon have a conversation about your wishes and needs regarding physiotherapy. What do you think the tasks of a physiotherapist are? |
| Are these also the tasks you want? If not, which tasks would you like a physiotherapist to perform? |
| Can you distinguish between your wishes and needs regarding these tasks? Which tasks do you need from a physiotherapist, and which tasks would you like to receive but don’t necessarily need? |
| Would you prefer the tasks of the physiotherapist to be directly focused on you, or can they also be directed at your surroundings? For example, should the physiotherapist provide advice to your caregivers or family on how to best support you? |
| Can you distinguish between your wishes and needs regarding these tasks? |
| What is your opinion on the role of a physiotherapist in treatment? Should the physiotherapist always be the guiding therapist, or can they also design a plan where others guide you during exercises? |
| Can you specify your wishes and needs regarding the role of the physiotherapist? |
| What is your opinion on the use of materials during physiotherapy treatment? (e.g., exercise equipment, homework exercises, illustrations, etc.) |
| Do you have any wishes or needs regarding the use of materials? |
|  |
| 2. Structure of the Treatment |
| If you perform exercises with a physiotherapist, do you prefer individual physiotherapy or group physiotherapy? Can you explain the basis of this preference? |
| How long do you think a physiotherapy session should last? Do you have any wishes or needs regarding the duration? |
| How often do you think you should receive physiotherapy treatment? Do you have any wishes or needs regarding the frequency? |
| Where should physiotherapy treatment take place, in your opinion? (e.g., the resident’s room, a shared space such as a living room or hallway, a gym, or outside in a garden or public space.) |
| Do you have any wishes or needs regarding the location of the treatment? |
|  |
| 3. Communication |
| What do you find important regarding communication with a physiotherapist? |
| Can you specify your wishes and needs regarding communication? |
| Are there certain communication techniques you find important when interacting with a physiotherapist? By communication techniques, we mean things like adjusting the speed and clarity of speech or the physiotherapist’s position relative to you (e.g., if you are lying in bed or sitting in a wheelchair). These are just examples. Are there specific communication techniques you consider important? |
| Can you specify your wishes and needs regarding communication techniques? |
|  |
| 4. Setting Goals |
| What is your opinion on setting goals in physiotherapy treatment? |
| Can you specify your wishes and needs regarding goal setting? |
| What is your opinion on involving the resident (in this case, yourself) in setting treatment goals? |
| Can you specify your wishes and needs regarding being involved in setting treatment goals? |
| What is your opinion on supporting “self-management” in physiotherapy? |
| What is your opinion on promoting self-management and healthy movement through a physiotherapist? |
| Can you specify your wishes and needs regarding supporting self-management? |
|  |
| 5. Involvement of Family Caregivers/Relatives |
| What is your opinion on involving family caregivers or relatives in physiotherapy treatment? |
| Can you specify your wishes and needs regarding involving family caregivers or relatives? |
| When do you think family caregivers or relatives could be involved in physiotherapy? |
| Can you specify your wishes and needs regarding when family caregivers/relatives can be involved? |
| How often do you think family caregivers or relatives could be involved in physiotherapy? |
| Can you specify your wishes and needs regarding how often family caregivers/relatives can be involved? |
